# Coping with uncertainty during healthcare-seeking in Lao PDR

**DOI:** 10.1186/1472-698X-13-28

**Published:** 2013-06-19

**Authors:** Helle M Alvesson, Magnus Lindelow, Bouasavanh Khanthaphat, Lucie Laflamme

**Affiliations:** 1 Department of Public Health Sciences, Karolinska Institutet, Widerströmska Huset, Tomtebodavägen, Stockholm, 18A 171 77, Sweden; 2 Brazil Human Development Department, The World Bank, SCN, Quadra 2, Lote A. Ed. Corporate Center, 7th andar, 70712–900 DF, Brasilia, Brazil; 3 Indochina Research Laos Ltd, IRL Building, 282/17 Phontong-Savath, PO Box 1887 Chanthabouly District, Vientiane Capital, Laos

**Keywords:** Healthcare-seeking behavior, Uncertainty, Maternal and child mortality, Qualitative interviews, Lao PDR

## Abstract

**Background:**

Uncertainty is regarded as a central dimension in the experience of illness and in the processes of alleviating it. Few studies from resource-poor settings have investigated this and how it interacts with other factors. This study aims to shed light on how healthcare-seeking develops in the context of multiple medical alternatives and to understand what bearing uncertainty has on this process.

**Methods:**

The study was conducted in six purposively selected rural communities in Lao PDR. In each community, two focus group discussions were held: first with mothers and then with fathers of children younger than five years old. Eleven in-depth interviews with caregivers of severely sick children were conducted. Subsequently, traditional healers, drug vendors, community health workers, nurses and medical doctors were recruited for interviews or group discussions. The data were transcribed and key themes and similarities were identified. Additional readings were conducted to better understand the interactions of factors during which uncertainty was identified as one of several factors mentioned during interviews and focus group discussions.

**Results:**

Care-seekers expressed a strong preference for initially seeking local providers. Subsequently, multiple providers were consulted to increase the chances of recovery. This resulted in patients leaving the health facilities before recovery and in ending the recommended treatment regime prematurely. These healthcare-seeking decisions reflect the social significance of being a responsible caregiver and of showing respect for household norms. In general, healthcare-seeking was shrouded in uncertainty when it came to selecting the right provider, the likelihood of finding the real cause of the illness, spending savings on treatments and ultimately the likelihood of recovery.

**Conclusions:**

Care-seekers’ initial strong preference for local providers irrespective of the providers’ legitimacy indicates the need for a robust primary healthcare system. Care-seekers’ subsequent consultations must be understood in the light of their uncertainty regarding the skills of the available providers. The social connotations of seeking healthcare including the vulnerability of poor households in public health facilities were taken into account to only a limited extent by health workers. Health workers should have greater awareness of the social and cultural aspects of seeking care.

## Background

### Healthcare-seeking and the role of uncertainty

The study of people’s use of and attitude towards healthcare alternatives is important for understanding constraints in healthcare utilization [1-3]. This is particularly important in communities where the determinants of healthcare-seeking are poorly documented. Community perceptions and practices are influenced by both global and local mechanisms. The globalization of biomedical drugs and commodities, for instance, has increased the available care options in urban and rural areas around the world during the last few decades [4]. Also, the co-existence of different healing practices [5], of popular, traditional, allopathic medicine in addition to self-care, is a characteristic of many health systems even though the actual availability of the different types of providers may vary [6,7]. Multiple social, individual and environmental factors affect the local patterns of healthcare-seeking. Apart from the level of individual knowledge about local illness categories and causes, important social and environmental factors include, among others, long distances to health facilities, lack of drugs, generational and gender-based constraints in deciding to seek care and medical costs [3,5,8,9]. In situations of ill-health, the possibility of not recovering is also a factor that people deal with and this can also affect their behavior and preferences when seeking care [10].

Uncertainty is a dimension of the experience of illness and the processes of alleviating illness that has been put forward as an additional important aspect of healthcare-seeking [11]. Uncertainty is a theoretical concept in a wide range of disciplines including not only medicine and nursing but also psychology and anthropology [12]. The anthropological entry point to uncertainty is that illness (along with other types of misfortune) demands action and evaluation of the consequences [10,13-16]. Uncertainty takes the study of illness back into the reality of people’s everyday life where they are seen as actors, not spectators, trying to alleviate suffering [17,18]. It means that guidance on uncertainty is conveyed in conversations with those around them and that the misfortunes and people’s responses to them are mediated by the values shared with others [11].

It has been argued that doubt and uncertainty in the process of seeking healthcare increase in conjunction with the rise in the number of treatments available and the growth in medical knowledge [19]. With the availability of multiple care options, a trial and error search of relief and cure is possible [11,20]. Uncertainty, as one factor influencing, engaging and affecting choices in healthcare-seeking [11,18,21,22], has for example been studied in doctor-patient interactions. Interestingly, research on physician disclosure of uncertainty as regards treatment alternatives and outcomes has shown higher patient satisfaction with the care received in a medical clinic in a high-income setting [19]; while uncertainty about the quality of life of preterm infants disclosed by physicians made medical decisions by caregivers related to the infant very difficult [23].

The landscape of care alternatives is diverse in orientation, in treatment and in the level of specialization. The outcome of treatments is often uncertain or poor in low-income settings. Maternal and child morbidity and mortality are still very high in many countries [24,25]. Progress in under-five mortality can be seen around the world but this progress is not equally distributed among regions [26]. The mortality of newborns, which makes up for 40 percent of under-five mortality, has only been marginally reduced during the last fifty years [27].

Focused on the healthcare-seeker perspective, the aim of this study is to increase knowledge about how people in low-income settings relate to the healthcare options available to them when maternal and child health is threatened and when the care can be provided by a variety of medical alternatives. We look at different factors influencing healthcare-seeking and pay special attention to how uncertainty comes into play in this process.

### Seeking care in Lao PDR

The study field is in Lao PDR, which has one of the highest maternal and child mortality rates in East Asia [28]. Studies of utilization rates of formal healthcare are few in the country and estimates indicate low and slightly decreasing rates (2003–2008) [29,30]. Outpatient care-seeking at formal providers is falling for all socio-economic groups except for the best-off, resulting in a widening inequality in utilization rates. The measles immunization rate at one year of age is 33 percent among the poorest 20 percent, and 60 percent among the wealthiest 20 percent [31]. The disease burden of children under five is high and communicable diseases are responsible for the largest proportion [32].

The reasons for low rates of utilization are manifold. Topographic conditions hinder large parts of the rural population from reaching a health facility. User fees have been increased and out-of-pocket payments account for almost three-quarters of the total national health expenditures in the country [33]. Both costs of travel and services are well-known barriers to health-seeking behavior especially for poor households [29]. Health insurance schemes are being implemented but coverage and quality of implementation is patchy and reaches mainly the well-off such as civil servants and employees in larger private companies [34].

The transition from a state-dominated health system into an increasingly mixed market accelerated in the early 1990s when private clinics and pharmacies were rapidly established in urban areas [35,36]. Mobile drug vendors from Lao PDR or from neighboring countries have been reported since the 1960s while spiritual practitioners (diviners among animistic groups and fortune-tellers among Buddhist groups) and herbal practitioners have been considered an integral part of way of life of the different ethnic groups since the beginning of these communities [37]. Private pharmacies and clinics represent the most recent types of providers [35]. Little is known about the scope or quality of informal providers but studies on licensed and unlicensed pharmacies indicate that there is considerable room for improvement in not only staff knowledge but also drug quality [38-40]. How these providers are perceived by users and the importance ascribed to biomedical drugs as a treatment option are poorly documented.

In describing a health system, the concepts of public/private health institutions and biomedical/traditional medicines are often used [41]. These distinctions are useful analytically but should be conceptualized on a continuum which allows for overlaps. This is especially true in Laos where both biomedical and herbal medicines are recognized as important ways of decreasing the burden of disease and traditional medicine is an integrated part of the Lao National Drug Policy [35]. The Government has shown commitment to improving health outcomes, in particular for maternal and child health, and the National Health Strategy emphasizes a better distribution of the health infrastructure, better performance of health workers and facilities in order to increase utilization rates [42].

Lao PDR is one of the world’s most linguistically and ethnically diverse countries with 49 ethnic groups officially recognized by the Government [43], which, in turn, from an anthropological perspective, can be divided into around 230 ethno-linguistic groups [44]. The groups belong to one of four main linguistic families: Lao-Tai; Mon-Khemer; Hmong-lu Mien; and Chine-Tibet. The ethnic Lao-Tai generally practice Buddhism while the majority of the non-Lao ethnic groups adhere to traditional animistic religious practices - even though there are variations in the local expressions and many of the religious practices show some degree of syncretism. The ethnic groups are also diversified in terms of livelihood and this ranges from hunting and gathering, different forms of swidden cultivation in the upland areas, to wet-rice farming in the lowlands. The social dimensions of healthcare-seeking are complex in Lao PDR due in part to the cultural and religious diversity. The Buddhist Lao-Tai-groups practice a matrilocal or bilocal residence pattern after marriage while the ethnic minority groups mainly follow a patrilocal pattern [44]. Lao Tai women thus live with their parents and relatives who help them to navigate in the social landscape during illness events better than patrilocal ethnic minority women. Nevertheless, family relations and respect for the older generation are very important across all groups. Even though respect for grandparents and in-laws is important, interactions between a sick child’s parents and grandparents does not automatically imply agreement on which actions to take. Coping with disagreements on where to seek care first was an aspect of healthcare-seeking that has been identified by parents who had lost a child [45].

## Methods

The data used herein were collected in the context of a broader study, which looked not only at patient and provider perspectives on healthcare-seeking behavior during fatal and non-fatal illness episodes but also at quality of care in public and private health outlets in Lao PDR. The study was conducted in rural communities of two provinces in the southern and central part of the country in February and March 2009 (for further details see [45]). In each of the provinces of Xekong and Savannakhet, we purposively selected one district. The two study districts have a total population of 55,000 and the population is ethnically diverse, including both ethnic minority groups, such as Levee, Alak and Oy, and Buddhist Lao Tai groups (65–75 percent) [46]. The Lao-Tai groups live traditionally in lowland areas, which are easier to access than the traditional habitat of minority groups residing in the upland or mountainous areas. The selected districts are in many ways typical of rural Lao PDR.

The districts were selected on the grounds that they had a functioning district hospital and some provision of private treatment from drug vendors or private doctors and nurses. These selection criteria reflect our objective of understanding care-seeker preferences when they in fact had several healthcare alternatives to choose between. Within each district, the data collection took place in six purposely selected rural communities of which four had “good access” (less than two hours walk) to a health center and two had “poor access” (more than four hours walk). The nearest health centers to each of these six communities were all included in the data collection and represented an equal mix of health centers with high and low outreach programs (measured in high and low immunization rates).

### Data collection

This study is based on the following materials. In each of all six villages, two Focus Group Discussions (FGD) were held; first with mothers and then with fathers of children younger than five years old with the purpose of: i) mapping out healthcare providers in the area and discussing the strengths and weaknesses of the compiled providers; ii) identifying five households who had experienced a fatal or severe illness episode during the last 24 months in children under five or in mothers; and iii) identifying factors that influence healthcare-seeking practices. Subsequently, representatives of traditional healers, drug vendors, Village Health Volunteers (VHV), Traditional Birth Attendants (TBA), nurses at the six selected health centers and medical doctors at two districts and one provincial hospital were included for interview or FGD (Table 1). It should be noted that the purpose of our study was not to define a distinct line of legitimacy among providers and when talking about drug vendors, we are referring to people who “operate on the margins of legitimacy” [47].

**Table 1:** Description of the demographics of the study sample

	**Women**	**Men**	**Caretaker during illness**	**Nurses**	**Doctors**	**VHV/**	**Drug vendors**	**Traditional practitioners**	**Total**
						**TBA**^**a**^			
Focus group discussions (participants)	6 (46)	6 (36)	-	8 (29)^b^	-	6 (43)^c^	-	-	26 (154)
Individual interviews	-	-	11	-	6	-	7	11	35

^a^ Village Health Volunteer (VHV) and Traditional Birth Attendant (TBA).

^b^ Nurses were selected from 2 district hospitals and 6 health centers. The names of the districts are kept anonymous since there is only one district hospital in each district and we are presenting data on delays in received care.

^c^ The VHV and TBA represented 22 different villages.

Out of 30 households, 11 had experienced a fatal health episode [45] and 8 interviews concerned an illness of the mother or obstetric complications (unpublished data) and 11 households had experienced severe illness in a young child. These interviews with caregivers of severely sick children are included in the analysis.

The fieldwork was carried out by spending one to three days in each community; interviews were performed in the homes of the families while the FGDs were held in a public but quiet location in the community. Interviews and FGDs with providers were carried out at the location of their services, which often provided opportunities for brief observations of the interactions with patients who sought care during the 1½ hours of the interviews. Two Laotian field workers with experience in qualitative data collection in rural areas, of whom one is also one of the authors (BK), led the discussions and conducted the interviews. All data collections were initiated with a presentation of the objectives of the study and informed consent was given verbally by the participants. They were accompanied by two note-takers and a field manager who assisted in liaising with the local authorities for permission to carry out the study and to mobilize participants.

### Data analysis

The FGDs and interviews were recorded, supplemented by notes from note-takers and moderators. Condensed transcripts (which excluded transcription of non-substantial clarifications between moderator and participants) were developed in Lao or directly translated into English by the two moderators before an analysis inspired by the interpretative description approach was carried out by HMA and BK [48,49]. The interpretative approach entailed repeated readings of the transcripts first by location of the data collection and secondly by type of participant while at the same time making notes on themes and similarities manually in the transcripts. For men and women, we grouped their perception of the strengths and weaknesses of each provider type in tables in order to compare views within and between communities. In a second step, these statements were compared to the challenges in health-seeking behavior listed by the providers interviewed. This data triangulation between the views of providers and care-seekers clarified some of the challenges in the interactions between them [50].

The in-depth interviews with caregivers and the parts of the FGDs that addressed experiences on specific healthcare-seeking processes and factors that influenced the process were analyzed in sequence, first by highlighting recurrent themes across many of the FGDs and interviews. This process identified factors related to (i) a preference for local providers; (ii) health costs as a constraint; and (iii) unease in interacting with health providers that were not located in the community. This left us with several factors and examples of constraints in seeking care that had not yet been analyzed in multiple transcripts. Secondly, through an iterative process of comparing these statements and experiences, HMA and BK identified more sub-themes that led into the significance of uncertainty in healthcare-seeking. This was a theme that we had not set out to explore and for this reason we did not prompt participants in FGDs or interviews on this dimension. However, during the analysis of the material, aspects of ambiguity and doubt were addressed by both fathers and mothers in FGDs, by caregivers to sick children and by some of the providers located in the communities. This made it possible to compare and contrast the ways in which dimensions of uncertainty during illness and ways of alleviating it were mentioned by participants.

Ethical approval for the study was obtained from the Lao National Ethic Committee for Health Research (N017/NECHR) of the Ministry of Health in Lao PDR.

## Results

We will first present how community members described the various health providers they consulted to set the stage for the analysis on the dimensions of factors influencing healthcare-seeking including uncertainty.

### The medical landscape in two rural provinces

Through the community-based data collection, we identified a high number of different provider types, each of which was used for different reasons. These dynamics will be presented below (Table 2). All villages mentioned the frequent consultation with VHVs, TBAs, herbal or spiritual healers and nurses at the health center. FGD-participants in the two remote villages listed fewer private and public providers than the villages with better access. All villages revealed discrepancies between the officially stated availability of providers and their actual experience.

**Table 2:** Types of health providers by perceptions of main services provided

**Provider**	**Provider in Lao**	**Main perceptions by FGD participant**	**Position in the health system**
Village health volunteer	*Aa saa sa mak saa tha la na souk ban* (*aw saw saw baw*)	Selling a limited number of medicines. Knowing a limited number of sicknesses.	Trained by MOH. Member in the Village Authority Committee
Trained birth attendant	*Pha doung khan*	Prenatal advice and assistance during home delivery.	Trained by MOH. Member of Village Authority Committee.
Traditional birth attendant	*Moh tam Nyae*	Helps during complicated births, changing the position of the baby during pregnancy.	Existed before the expansion of primary healthcare.
Herbalist	*Moh Ya*	Produces, sells and treats with herbs most often found in the local forests.	Active both among Buddhists and animists. Recognized as a traditional provider by the MOH
Diviner	*Moh Phi*	Divination (communication with ancestral and nature spirits) as tool to identify cause of illness and treatment. Can determine if illness has a spiritual cause or not. Can only treat illnesses with spiritual cause.	Most popular among animist groups. Not considered integral to the formal healthcare system
Fortune-teller	*Moh duo*	Divination (communication with Buddha to identify cause of illness). The fortune-teller will organize the ceremony /treatment in the room with the Buddha image, plate of candles and flowers (set up the room like temple). Uses birth dates, times and Chinese horoscope to identify cause of illness.	Most popular among Buddhist clients. Not considered integral to the formal healthcare system
Nurse (retired, military trained or after formal work hours)	*Thaan moh* (*Phaed khao*, *Phaed tha haan lou phaed noak mhong kaan*)	Can diagnose, provide treatment and sell medicine. Have few medical instruments such as stethoscope.	Most frequently trained in the military and also can offer mobile services. Some of these nurses are also civil servants working at the health center or district hospital; but the most common is that they have retired from the civil service or the military. This is extra income for the nurses.
Mobile drug vendor	*Moh ya kheuan ti*	Known for selling strong and effective medicines. They normally only sell medicines and offer treatments of for example joint pain.	Often they will live in the same district or same village and travel to nearby villages on a regular basis. Will often offer his services from the small retail shop in the village. Only encountered male drug vendors. Have some type of medical background as a VHV or the military. This is their main business.
Mobile Chinese/Vietnamese drug vendor	*Moh ya chin kheuan ti*	Known for selling strong and effective medicines. Most frequently active close to border areas.	Will visit village by village in the same province. Often travel by motorbike and can reach remote areas. Sometimes they sell other products like cooking pots. Often arrives after the harvest period.
Nurse at health center	*Phaed souk saa laa*	Few medical types of equipment available and few diagnostic procedures performed. Uncertainty whether nurses are able to diagnose more diseases if they have the instruments; or if the MOH did not provide them with more tools due to their limited training or experience. Limited number of drugs for sale.	There is now only one training level of nurses (3 years of training). Earlier a nursing diploma was achieved after 1–2 years of training.
Nurse and doctor at district level hospital	*Pha Nyaa baan lae thaan moh hong moh meuang*	Varied number and quality of diagnostic procedures, type of equipment and treatments of illness.	
Nurse and doctor at provincial, central, military hospital	*Pha Nyaa baan lae than moh hong moh kheang*	Broad access to diagnostic equipment and treatments including surgery. Good quality of care	
Private pharmacist or staff in pharmacy	*Haan khay ya aek ka sonh lue paed yu haan khay ya*	Selling medicines based on description of symptoms, prescriptions or sometimes measurements.	Owned by a medical doctor or pharmacist. In the rural areas there are class 3 pharmacies that sell drugs from the market place or in front of the hospital. Majority have permanent premises from which they sell medicines.
Private clinic	*Kri nik aek ka sonh*	Broad range of diagnostic equipment, strong medicine and well performing health staff.	
Private hospital in Thailand or Vietnam	*Hong moh aek ka sonh thai lue hong moh aek ka sonh viet nam*	Full range of diagnostic equipment, treatments and professional and qualified health staff	

The list of providers reveals a mix of healthcare-seeking at both authorized and unauthorized providers. Private providers are limited in numbers and coverage as was also indicated by community members in remote villages who had used a limited number of private care options. Some of the private providers were retired nurses or military nurses.

### Healthcare delivery: constraints in accessing a fluid and changing landscape

When it comes to seeking care, interview data concerning the severe illness of ten children indicated that health centers were the most frequently reported source of care sought. However, none of the households ascribed recovery to the services received. Four of ten episodes implied admission to a provincial hospital either in Laos or in Thailand, which indicates the severity of the cases. Poor families and girls as patients were few, however. Data in FGDs pointed to VHVs and informal providers in the communities where people live, as the most frequently consulted providers in addition to self-treatment involving herbal and biomedical medicines. Another study in Laos found public providers had only been consulted in 4 percent of the identified illness episodes during the last two weeks [51]; and a larger survey found that 18 percent of individuals who had been sick during the last 4 weeks had consulted a public provider [30].

Travel time to the different providers was a factor that influenced the healthcare-seeking behavior. Having to carry a sick child or mother even over shorter distances is a real impediment for families in remote rural areas. It was also evident that community members paid attention to the de facto availability of providers and to staff changes, which made decision-making more difficult especially during the distress of an ongoing disease during the weekends. The fluidity of healthcare was exemplified in the transfer of an experienced nurse at the health center; of a mobile drug vendor who was starting to visit the village two or three times a month instead of once; or of a herbalist having a more limited number of herbal treatments available than he used to. VHV, TBAs and spiritual diviners were the providers described as the most stable. The changing landscape of providers was discussed among neighbors and relatives but also occasionally at community meetings with the Village Authorities (VA).

We also identified some overlaps between the provider types. The best-known are nurses employed at hospitals or at health centers who provide services in their free time as well; and medical doctors working at a hospital in addition to a private clinic often in his/her home. Two nurses in a FGD explained:

Nurse 1: We treat some patients [after hours] but not many, most are close relatives. When they get sick they call us to give an injection. We can treat them, but it depends on the symptom - if the sickness is serious we tell them to go to the hospital…. I bought the medical instruments such as thermometer, blood pressure manometer out of my own pocket.

*Nurse 2: The reason patients come to see me at home is because they are busy with their families. When I go to their houses I will not charge them much; for example paracetamol is 5,000 kip [$ 0.6] per one injection. In addition, they will give me money to pay for a liter of petrol* (Female nurses at a health center).

In a FGD with nurses, they commented on the doctors as follows:

*The doctor at the clinic is the same as the doctor in the hospital. The doctor in the hospital normally opens his own clinic at home after work. If the clinic cannot treat the patient because they don’t have the right equipment, they send the patients to the hospital* (Female nurse at a district hospital).

Medical doctors acknowledged these practices in interviews and added that the informal services are more pronounced in urban areas where the demand is higher. Another type of overlap was regarding the services provided by VHVs. In some of the villages, they were providing injection services even though formally this is not included in their services. It also occurred that VHVs ceased their formal association with the MOH but continued to provide treatment and to sell drugs in the community similar to the services of drug vendors. A drug vendor gave an example of his services:

*Some patients were diagnosed by the nurses at the hospital; they were, for example, told that the patient was suffering from malaria. The patient had to stay overnight at hospital but they did not want to be admitted which is quite typical in this area. So they come to me instead. In addition, they didn’t want to spend more money so some clients bought a drip bag solution at the hospital and then let me administer it for them* (Male drug vendor in rural area).

The MOH recognizes herbal medicine as an effective line of treatment and in some FGDs with nurses they confirmed that they refer patients to licensed herbalists for complementary treatments. At the house of VHVs or TBAs, it also occurred that the older woman or man in the family was a diviner and they would treat the same patients with their different methods when needed. We found few other examples of local providers having contact with each other or referring patients to each other, however.

### The preference for local providers minimizes uncertainty and costs

Discussions on provider alternatives and on sequencing healthcare during specific illnesses - with mothers and fathers to children under five and with providers - illuminated the doubts and ambiguities that lingered during healthcare-seeking. There was a clear preference for seeking local providers as a first response to illness in all FGDs. Eight of the ten households interviewed about a severe illness had consulted a traditional practitioner, drug vendor or VHV in the village as their first response and for two households living in a community with a HC, the HC-nurses were the first providers contacted. This pattern was confirmed in the FGDs with caregivers. The attractiveness of first seeking care within the community had several aspects as these two mothers explained in a focus group:

R2: If we talk about who we trust more when taking medicines then we will choose the nurses at the health center because they have some medical training and the center is located close to our village as well and we know them better and thus, we can ask them many questions. In addition, they allow us to have the treatment and pay later.

*R4: We can pay later at the health center and the nurses at the health center have their own medicines for which they’ve paid for out of their own pocket in case there are no medicines funded by the government available. We can also pay in kind with ducks, chickens or pigs instead of money. We like the services at the health center* (Mothers in a village with good access)*.*

Doctors recognized the popularity of informal services in the communities:

*The people in the rural area like to have the treatment that makes them recover quickly and they don’t care what the side effect from that treatment is if the medicine is too strong. Many people here like to be treated by the illegal nurse who is a former nurse and travels to treat people in the villages or calls on the patient to provide the treatment at his/her house. These illegal nurses prescribe too strong medicines: the patient has only a cold, they prescribe an antibiotic and the patient recovers. So the villagers believe that she/he can treat better than the hospital* (Male doctor at district hospital)*.*

Another appreciated aspect of local provision of healthcare was to be diagnosed with familiar diseases. Many illnesses among children started with perceived normal fever, cough or diarrhea. The parents knew that the VHV or drug vendor had a limited amount of drugs available for sale but they were also aware that the local providers only know of a limited number of diseases. These familiarities favored the consultations with local providers. In contrast to well-known fevers, unknown symptoms resulted more often in direct consultations at the district or provincial hospital. The grandparents of a 15-month-old boy described the grandson’s severe illness:

*My grandson became sick. In the middle of the night he had a temperature, cold feet and diarrhea. I had never seen these symptoms before. Next morning I took him to [the district] hospital. I did not take him to the HC because they do not have a variety of medicines or of equipment. It would have wasted our time to seek care there. We were very worried about his illness; we were afraid he would die* (Grandfather in village with good access)*.*

The ambiguity of the combination of symptoms was most likely one of the determining factors for seeking care promptly. Other factors influencing the decision in this particular case were economic ability to pay for the treatment by the grandparents and that the grandparents were the single decision-makers since the parents of the child were working in the capital at the time of sickness.

For severe illnesses, caregivers explained in FGDs that they resulted primarily in visits to the district hospital and less frequently to the provincial hospital. Hospital consultations and especially admissions were experienced as being linked with high costs of medicines, fees, transportation in addition to the costs of accommodation for caregivers. One mother explained in a focus group:

*When we are told the patient has to stay overnight at the hospital we agree with that. But the problem is that we have no money; who will assist in doing the housework, who will take care of the patient on a daily basis and how can we get food to eat [at the hospital]?* (Mother in village with good access)*.*

A VHV talked about her own experiences as a patient:

*I have been to the provincial hospital; they have modern equipment, doctors with advanced degrees, and many specialists. But if we want to go there we have to sell cattle; for our 200,000 kip in cash [equivalent to US$ 25] will only pay for the travel costs. We prefer the place that has everything to support our treatment but we don’t have that much money so we cannot choose what we like* (Female VHV in village with low access)*.*

The lack of predictability in how much the treatment would eventually cost was a very serious concern. When receiving inpatient care, the medical costs are expected to be paid on a daily basis. Information about the maximum costs of the complete treatment could not be known. Caregivers were very worried that they would run out of money before the sick family member had recovered since it was well known that without payment at the hospital, treatments would be discontinued. Some herbalists have, by contrast, a payment policy that minimizes the risks of depleting savings without finding a cure. A small fee is initially paid for the herbs and only upon recovery of the patient will a larger fee be requested.

### The challenge of identifying the causes of disease and deciding which provider to go to

Although leaving the hospital early was clearly rational from an economic point of view, it was also a response to the availability of other treatment options. When asked about their tasks during children’s illness episodes, the fathers in FGDs answered that it was their “*responsibility to find the right provider*”. This emphasis on identifying “*the right provider*” as one of several important tasks, which also include finding transportation and payment, is one of the answers that led the analysis into the pragmatics of uncertainty. In addition, in the ten cases of child survival, irrespective of which provider was first consulted, the child did not recover at once and several other providers were consulted. The second consultation was with a different provider in all households. In three cases, the same provider (VHV and HC) was subsequently consulted twice. The pattern of continuously seeking a new provider was well recognized by all types of providers. One VHV said:

*In some cases, people in our village take medicines from this place [VHV] for 3 days and recover; but 5 days later when they get sick again, they seek treatment in another place. Another example is that some people come back home while they are being treated at the district hospital. They did not recover completely but they had to come home because they were concerned about their houses. After 2–3 days they got sick again, but instead of going back to the district hospital, some went to the neighbouring district hospital, and some were treated by the VHV in the village. If they still did not recover, they went on to the provincial hospital* (Male VHV in village with good access)*.*

Seeking the opinion of a second provider, rather than revisiting the first one seemed to encompass different rationales. One was on the grounds that finding the cause of the illness is a difficult task that may require several attempts, which was thought to improve the prospect of recovery. A second had to do with the expectation that any first consultation would imply that the provider would do his/her best and prescribe his/her best medicine. One father, whose son was diagnosed with malaria, received treatment and recovered from this episode, explained:

*Most of the times I will not come back to the previous providers; I will seek new providers all the time because there is no need to go back to the same provider if it does not result in recovery….. [In case with child] we were impatient and afraid he had a serious fever that would be difficult to cure. Therefore we did not care about medical expenses….. But, if he had had a fever again then we would not have gone back to the same provider; we would seek other providers and they would try out new medicines* (Father in village with good access)*.*

Also when the illness episodes had extended over several weeks with periods of improvement and relapses, the parents would continuously seek new providers. A mother described her concerns during the admission of her young daughter at the district hospital:

*Most often the health staff there will give the patient an injection as soon as he/she arrives at the hospital and let the patient lie on the bed and wait about 3–4 hours; after that they will come to the patient again and ask about the symptoms. This time [with my daughter] we were very concerned about our child; we wanted to know if she would recover or not. If not, we would take her to other places* (Mother in village with good access)*.*

The hope of successful treatment from new providers is not only relevant in evaluating biomedical providers but also divinations with spiritual healers. There seems to be a notion that a diviner’s diagnosis is not guaranteed to be complete the first time, which will warrant consultations with other diviners or with other providers. It is of note that the practice of seeking multiple providers created some frustration among public health workers. A VHV described his concerns in a FGD:

*Some people have treatment from several health providers such as illegal health providers, religious healers and VHV, but they do not do so continuously. They only spend a short time with each of them. Then they complain that they spent so much money for the treatment* (Male VHV in village with low access)*.*

### Household- and community-based familiarity with the provider minimizes uncertainty

It was largely agreed during the FGDs with mothers and fathers that the uncertainty involved in seeking care was better dealt with when the family, friends or neighbors knew the intended provider. At a HC or, for the communities located sufficiently near, at a district hospital, it was described as relatively easy to get to know the nurses working there and learn how they treated patients. But getting acquainted with a nurse was more difficult in provincial hospitals or for patients in remote areas. One strategy was to mobilize friends or relatives who knew health workers. The friends served many different roles such as prompting the decision to seek care; helping the caregivers through the registration at the facility and getting in contact with the health provider they knew; asking the doctor or nurse questions and in a general manner reducing the uncertainty of not being well taken care of at the hospital. Among the 10 families interviewed on a severe episode of their child, the four families who had consulted the provincial hospital had all received help from relatives or friends when first seeking hospital care. The need of normative and linguistic support, especially to non-Lao speakers, was confirmed in discussions with nurses, doctors and VHVs. Two nurses explained:

Nurse 1: Non-Lao speaking patients are very nervous when they are at the hospital. It may be because they are afraid to talk to the doctor and they may be afraid that nurses and doctors will get angry if they don’t understand what the doctor says.

*Nurse 2: I am from an ethnic minority and I can speak my minority language. I think it is quite important that nurses have the same background as the patient because the patients will talk to you in more detail about their symptoms, moreover, we understand each other in terms of culture and customs* (Female nurses in rural remote village)*.*

### Social connotations of care affect the process of seeking health providers

Healthcare decision-making activated the social roles of parents and household heads and the actions taken reflected how well they handled their role as parents and as respectful members of an extended family. A grandfather taking care of his daughter’s two-year-old child explained decision-making in his household:

*The parents of the children make the decision. The parents discuss with each other first and then they consult with me together with relatives; we, for example, discuss when the provider lives far away from our home because of high costs and expenses. If it is about seeking care at the dispensary or village health worker, the husband and wife can make the decision by themselves* (Grandfather in village with good access)*.*

Men and women agreed that the risk of high medical costs implied different decision-making processes to those implemented prior to low-cost consultations with local providers. It was, in particular, clear that with better access to health facilities, women made more healthcare-seeking decisions when their children fell ill. As a consequence, mothers, who had little control over the financial resources in the household, and/or lived close to an HC, could also seek care by themselves while women in the remote villages only consulted the VHV or TBA after involving other family members. Drug stores, licensed or unlicensed, provided an opportunity for mothers to act fast without involving other family members. Most women do not travel alone to district or provincial hospitals and even though a child was recognized as being in need of treatment, it was considered very important to comply with this practice. On the other hand, it was stated that healthcare decisions and the need to involve relatives were increasing since the medical costs of public healthcare had been added to the burden of medical expenses of traditional treatment. Disagreements in the group of relatives making the decisions on healthcare were described as increasing. A mother in a FGD explained:

*I let my husband choose because he must look for the money - I cannot - and therefore, if he makes the decision to go to provincial hospital then he will look for money or borrow from his relatives. But in the past, we never had different ideas because we did not have any other options than the VHVs, the old midwife and the nurses* (Mother in village with good access)*.*

Healthcare-seeking also accentuated health workers’ expectations on community members. The interactions and type of relationship between care-seekers and providers was a topic that emerged at different occasions during the data collection. We have touched upon the reluctance of care-seekers to ask too many questions during consultations at public facilities. Local providers were perceived to offer a more accepting attitude to questions from care-seekers independently of the dignity of the question. Drug vendors were perceived to have good communication skills and were in general described as being better at explaining the diagnosis of patients while nurses tended to simply write a prescription. A TBA described how the actions and responses between providers and patients could go wrong at the public facilities:

*The government health staff do not often explain clearly to the patients about the benefits of medicines: why do they use these kinds of medicines for the treatment; how many kinds of medicines can be used for the same kind of disease; which have a better effect. They do not talk about this. They prefer to provide the treatment step by step and use the medicines that they have available. This makes people wonder if the patient will recover or not and they think that the health staff at the government hospitals are not as good as at the private clinic* (Female TBA in village with low access)*.*

Care-seekers were concerned that their poverty could influence the way they were treated at the hospital. A mother formulated this concern very clearly in a FGD:

*The health staff normally see that we are common people from the countryside but they still collect money from us for accommodation; for the treatment we pay at the same rate as the government officials whose employers pay for part of their treatment. I think the government should allocate some budget for the treatment of the poor* (Mother with good access)*.*

These community members had benefited from using better quality care options in Thailand during frequent working periods, which can have influenced their view on what good care in fact could be. Drug vendors were generally perceived to communicate in a more comprehensible manner and they also offered popular injection medicines, which drug vendors confirmed was an important part of their services. The skill to inject is an indication of quality in many settings [52] and the popularity of the injections posed a dilemma for VHVs who officially are not allowed to inject. The good communication skills of drug vendors however led to some concern. While participants worried that formal health workers are mainly driven by complying with official duties, there was some suspicion that informal providers sold them the most expensive rather than the most effective drug. The risk of harmful drugs was only mentioned in relation to purchasing drugs from mobile drug vendors who were not from the area. Especially in the FGDs with fathers, it was mentioned that there was no control over the safety of the patient once the mobile drug vendor had left the village.

## Discussion

We have outlined the pragmatic ways in which healthcare-seeking is affected by multiple and interlinked factors. Community members expressed uncertainty about selecting the right provider, about the likelihood of finding the real cause of the illness, about spending savings on treatments and ultimately about the likelihood of recovery.

High levels of out-of-pocket payments influenced utilization rates of public healthcare. Illness in Laos can imply financial emergencies for poor as well as for better-off households. This has also been demonstrated in other studies on coping strategies during illness [33,53]. A study in Vietnam found that among uninsured populations (similar to the populations in this study), the risks of catastrophic health expenditures are of great concern compared to the frequent but low-risk illness episodes [54]. In our study, the lack of information of the total medical expenses for a hospital admission was an additional constraint that negatively impacted healthcare-seeking. The lack of affordable public healthcare increases the appeal of informal providers who complement the services of the health centers and hospitals but at lower costs in the communities.

Aside from costs and convenience, the popularity of community-based services also depended on factors that were related to bridging the asymmetrical social and medical knowledge between patients and public providers. Some of the drug vendors functioned as interpreters, not only of language, but also of the meanings that were ascribed to drugs, of how slow or fast the body reacts to treatments, and of finding the right way of communicating during consultations. Establishing trust with providers was also very important for care-seekers and it was perceived that this was easiest to accomplish with local providers, including nurses at health centers. Trust between provider and patient is essential to provide good and effective care [55], to increase utilization rates [56], and to avoid exploitation [57].

Many studies on trust and provider-patient relationships have intriguingly been void of an analysis of the power relations involved [58,59]. Raising awareness among professional health workers about the asymmetrical relationship between provider and patient could address some of the sources of uncertainty of patients and potentially make consultations less intimidating for patients. In a study from Vietnam it was found that the lack of awareness of the values and attitudes of the majority culture in the health system inadvertently functioned as a barrier to minority patients [60]. By approaching consultations as social encounters, in addition to just medical ones, health workers could get a better understanding of the ways in which users experienced primary healthcare [13]. This could be important to address in the Lao health system through increased emphasis on provider-client communication. Key priority areas for improved responses from providers to patients include information about the danger signs of illnesses [45] and a justification of the chosen treatments including expectations on the timeline of recovery. Giving communities the opportunity to provide feedback on the quality of services received at the health facilities is another way to integrate experiences and perspectives of healthcare users into health systems.

In this study, the distinction between public and private providers was blurred by the fact that health workers were or had been civil servants who were now offering services informally. Parents focused on access to diagnostic procedures and drugs; and less on the formal training of the health workers. It has been found that awareness of drug quality among users in Lao PDR is limited [36,39]. The educational background, knowledge and perceptions of staff working in private pharmacies have been studied [39,61] and the moonlighting of civil servants including health workers has been found to influence their availability at health facilities in one study [62]. The recent substantial increase of the salary of civil servants including health workers is a positive development that can potentially reduce the economic need for health workers to work in multiple practices, but it has to be combined with other management reforms.

Alternative ways of distinguishing between providers are based on the location of the care, on gender, on type of treatment such as injection or oral therapy, and ailments of ambiguity and/or certainty [41]. In our study, the distinctions between familiar-unfamiliar symptoms and between familiar-unfamiliar health providers were in particular important and care-seekers approached each other as local experts to identify the best provider. Local exchange of medical knowledge is one important dimension of the ways in which people are driving and not simply benefiting from the health system [58,63]. The implication of these findings for the current primary healthcare system is that there is potential benefit in building stronger linkages between communities and health facilities. Horizontal mobilization of communities to implement evidence-based interventions targeting maternal and child health is acknowledged as an effective way to increase demand for healthcare in multiple settings [64,65]. The engagement of community health workers and volunteers with an interest in the health of the community and a definition of the roles and responsibilities of local health facilities are building blocks for improved interactions between communities and facilities [66]. Communication and counseling activities to bring about behavioral change in mothers with children under five improve the entry- and continuous contact-points between communities and a weak health system. This has not yet been implemented at full scale in Laos. In addition, mobilization or outreach activities to encourage peer education among husbands, in-laws and other persons in the extended family are an additional way to influence healthcare-seeking [67,68].

The findings do not imply that provider pluralism is negative in terms of health outcome but that the way to a cure in communities can be complicated. Leaving public health facilities before recovery (including avoiding admittance or referral) had strong financial rationales in our study but also indicated that care-seekers had doubts about the choice of treatment. Care-seekers were not only evaluating their choice in the light of traditional beliefs or ancestral obligations but also in the light of the response they received from the chosen providers. When a treatment was tried out, care-seekers were not convinced that it would work. Lack of perceived quality of care has been identified as a barrier to utilization of public health services [68,69]. Improving the quality of public healthcare services in rural areas is one way of reducing the increased risk of maternal and child mortality in rural areas [32,43]. There are good reasons to believe that the persistent mortality can be reduced; higher coverage of existing interventions for nutrition and disease prevention can reduce mortality for 0–3 year olds by around 25 percent [64]. Expansion of basic public health and nutrition interventions such as immunization, promotion of breastfeeding, Vitamin A supplementation and safe drinking water is a common characteristic of countries that have succeeded in reducing maternal and child mortalities the most [70].

The findings indicate that it is important to provide room for uncertainty in the analysis of health-seeking behaviors [11]. When we map care-seekers’ pathways of care, we should also embrace the alternative care options that were considered but rejected for different reasons. We should be careful not to expect clear-cut patterns of behaviors when the health options are far from clear-cut.

Care-seekers’ disappointments with failed treatments were not exclusive to the public providers but were experienced after consultations with traditional and private providers alike. The health system in the study area was negatively affected by environmental problems leading to greater difficulties in finding the needed diversity of herbs for medicine, shortage of health workers and difficulties in financing the health system. It was a challenge for the public health providers to profile themselves as essentially different from drug vendors, which indicates the fragile state of the health system. The strong preference for local provision of healthcare should be seen in this light. The increasing emphasis on the importance of quality of care also in weak health systems is an important contribution to the development of consumer rights in resource-poor settings [71,72] and also helps to minimize some of the uncertainty that originated in seeking care at facilities that care-seekers knew were not the best available.

### Limitations of the study

We attempted to minimize the association between the field team and the Ministry of Health during the fieldwork by emphasizing our interest in traditional and informal providers and by stressing the objective of understanding community members’ preferences. Nevertheless, the preference for informal providers is estimated as being underrepresented due to the short stay in each community. Self-treatment and non-treatment were not explored to the same extent as provider-based treatment and it is therefore likely that we underestimated their importance. Participant observation during ongoing illness episodes could illuminate more detailed considerations of alternatives in seeking care than we were able to in this study.

## Conclusion

Understanding the complexity of human behavior in healthcare-seeking is important for improving health practices. Care-seekers’ strong preference for local providers irrespective of the providers’ status of legitimacy indicates the need for a strong primary healthcare system. The increased number of types of available providers, without one standing out as providing distinctively better quality, fuelled uncertainties related to finding the right provider. To minimize uncertainty, care-seekers maximized their chances of finding a cure by consulting multiple providers. This pragmatism reflects the hope that one of them will work even though it is acknowledged that finding a cure is often complicated and uncertain. The normative connotations of seeking healthcare, which was one source of uncertainty for care-seekers, were little reflected by public health workers, which they, in this culturally diverse setting, should be prepared for. Community-based maternal and child health programs offer effective ways of strengthening the linkages between communities and primary healthcare.

## Competing interests

The authors declare that they have no competing interests.

## Author’s contributions

The study was conceived and designed by HMA and ML. Data were collected and analyzed by HMA and BK. All authors made contributions throughout the process of writing the manuscript and have read and approved the final manuscript.

## Pre-publication history

The pre-publication history for this paper can be accessed here:

http://www.biomedcentral.com/1472-698X/13/28/prepub

## References

[B1] Hausmann-Muela S Muela Ribera J Nyamongo I Health-seeking behaviour and the health seeking response DCPP Working Paper No 14 2003 http://www.dcp2.org/file/29/wp14.pdf

[B2] WHO Systems Thinking for Health Systems Strengthening 2009 Geneva: WHO

[B3] Claeson M Waldman RJ The evolution of child health programmes in developing countries: from targeting diseases to targeting people Bull World Health Organ 2000 78 10 1234 1245 11100618 PMC2560618

[B4] Whyte SR Geest S Hardon A Social lives of medicines 2002 Cambridge: Cambridge University Press

[B5] Janzen JM The quest for therapy in lower Zaire 1978 Los Angeles: University of California Press

[B6] Van der Geest S Whyte SR The context of medicines in developing countries. Studies in pharmaceutical anthropology 1988 Amsterdam: Het Spinhuis

[B7] Timmermans S Almeling R Objectification, standardization, and commodification in health care: A conceptual readjustment Soc Sci Med 2009 69 1 21 27 10.1016/j.socscimed.2009.04.020 19464781

[B8] Barnes-Josiah D Myntti C Augustin A The "three delays" as a framework for examining maternal mortality in Haiti Soc Sci Med 1998 46 8 981 993 10.1016/S0277-9536(97)10018-1 9579750

[B9] Gabrysch S Campbell OM Still too far to walk: literature review of the determinants of delivery service use BMC Pregnancy Childbirth 2009 9 34 10.1186/1471-2393-9-34 19671156 PMC2744662

[B10] van der Sijpt E Marginal matters: pregnancy loss as a social event Soc Sci Med 2010 71 10 1773 1779 10.1016/j.socscimed.2010.03.055 20627499

[B11] Whyte SR Questioning misfortune. The pragmatics of uncertainty in eastern Uganda 1997 Cambridge: Cambridge University Press

[B12] Penrod J Refinement of the concept of uncertainty 2001 10.1046/j.1365-2648.2001.01750.x 11430286

[B13] Mogensen HO Finding a path through the health unit: practical experience of Ugandan patients Med Anthropol 2005 24 3 209 236 10.1080/01459740500182659 16081334

[B14] Kleinman A Kleinman J Suffering and its professional transformation: toward an ethnography of interpersonal experience Cult Med Psychiatry 1991 15 3 275 301 10.1007/BF00046540 1935180

[B15] Einarsdottir J Tired of weeping: mother love, child death, and poverty in Guinea-Bissau 2004 Madison WI: University of Wisconsin Press

[B16] Good BJ Medicine, rationality and experience. An anthropological perspective 1990 Cambridge: University Press

[B17] Honkasalo ML Lindquist J An interview with Arthur Kleinman Ethnos 1997 62 3 107 126

[B18] Obermeyer CM Risk, uncertainty, and agency: culture and safe motherhood in Morocco Med Anthropol 2000 19 2 173 201 10.1080/01459740.2000.9966175 11307571

[B19] Gordon GH Joos SK Byrne J Physician expressions of uncertainty during patient encounters Patient Educ Couns 2000 40 1 59 65 10.1016/S0738-3991(99)00069-5 10705065

[B20] Ryan G What do sequential behavioral patterns suggest about the medical decision-making process? Modeling home case management of acute illnesses in a rural Cameroonian village Soc Sci Med 1998 46 2 209 225 10.1016/S0277-9536(97)00151-2 9447644

[B21] Feierman S Explanation and uncertainty in the medical world of Ghaambo Bull Hist Med 2000 74 2 317 344 10.1353/bhm.2000.0070 10863831

[B22] Anthropologists EAoS Uncertainty and disquiet 2012 France: Uncertainty and disquiet

[B23] Einarsdottir J Emotional experts: parents' views on end-of-life decisions for preterm infants in Iceland Med Anthropol Q 2009 23 1 34 50 10.1111/j.1548-1387.2009.01036.x 19449711

[B24] Bhutta ZA Chopra M Axelson H Berman P Boerma T Bryce J Bustreo F Cavagnero E Cometto G Daelmans B Countdown to 2015 decade report (2000–10): taking stock of maternal, newborn, and child survival Lancet 2010 375 9730 2032 2044 10.1016/S0140-6736(10)60678-2 20569843

[B25] Barros AJ Ronsmans C Axelson H Loaiza E Bertoldi AD Franca GV Bryce J Boerma JT Victora CG Equity in maternal, newborn, and child health interventions in Countdown to 2015: a retrospective review of survey data from 54 countries Lancet 2012 379 9822 1225 1233 10.1016/S0140-6736(12)60113-5 22464386

[B26] You D Jones G Hill K Wardlaw T Chopra M Levels and trends in child mortality, 1990–2009 Lancet 2010 376 9745 931 933 10.1016/S0140-6736(10)61429-8 20851244

[B27] Målqvist M Who can save the unseen? 2010 Studies on neonatal mortality in Quang Ninh province Vietnam: Uppsala University

[B28] WHO Country Statistics Global Health Observatory Data Repository 2012 http://apps.who.int/ghodata/?vid=11900&theme=country

[B29] Patcharanarumol W Health Care Financing for the Poor in Lao PDR 2008 London School of Hygiene and Tropical Medicine: Health Policy Unit, Department of Public Health and Policy

[B30] World Bank Out-of-pocket spending and health service utilization in Lao P.D.R. Evidence from the Lao Expenditure and Consumption Surveys 2010 Vientiane: The World Bank

[B31] WHO Lao People's Democratic Republic: health profile 2012 http://www.who.int/gho/countries/lao.pdf23761587

[B32] WHO Country Cooperation Strategy at a glance 2013 WHO http://www.who.int/countryfocus/cooperation_strategy/ccsbrief_lao_en.pdf

[B33] Wagstaff A Lindelow M Are health shocks different? Evidence from a multi-shock survey in Laos Policy Research Working Paper Series. Volume 5335 2010 Washington DC: World Bank

[B34] Zeluf G Perspectives, perceptions and experiences of community-based health insurance among different actors in Lao People's Democratic Republic 2011 Karolinska Institutet: Department of Public Health Sciences

[B35] Syhakhang L The quality of private pharmacy services in a province of Lao PDR: perceptions, practices and regulatory enforcements 2002 Karolinska Institutet: Department of Public Health Sciences

[B36] Syhakhang L Lundborg CS Lindgren B Tomson G The quality of drugs in private pharmacies in Lao PDR: a repeat study in 1997 and 1999 Pharm World Sci 2004 26 6 333 338 10.1007/s11096-004-0558-3 15683103

[B37] Tambiah SJ Buddhism and the spirit cults in Northeast 1970 Thailand: Cambridge Studies in Social and Cultural Anthropology

[B38] Sihavong A Lundborg CS Syhakhang L Kounnavong S Wahlstrom R Freudenthal S Community perceptions and treatment-seeking behaviour regarding reproductive tract infections including sexually transmitted infections in Lao PDR: a qualitative study J Biosoc Sci 2011 43 3 285 303 10.1017/S002193201000074X 21211093

[B39] Syhakhang L Freudenthal S Tomson G Wahlstrom R Knowledge and perceptions of drug quality among drug sellers and consumers in Lao PDR Health Policy Plan 2004 19 6 391 401 10.1093/heapol/czh054 15459164

[B40] Stenson B Syhakhang L Lundborg CS Eriksson B Tomson G Private pharmacy practice and regulation. A randomized trial in Lao P.D.R Int J Technol Assess Health Care 2001 17 4 579 589 11758301

[B41] Leach MA Fairhead JR Millimouno D Diallo AA New therapeutic landscapes in Africa: parental categories and practices in seeking infant health in the republic of Guinea Soc Sci Med 2008 66 2157 2167 10.1016/j.socscimed.2008.01.039 18314240

[B42] Ministry of Health Strategy and Planning Framework for Integrated Package of Maternal, Neonatal and Child Health Services 2009–2015 2009 Vientiane: Ministry of Health

[B43] Messerli PHA Epprecht M Phonesaly S Thiraka C Minot N Socio-economic atlas of the Lao PDR. An analysis based on the 2005 population and housing census Swiss National Center of Competence in Research 2008 North–south, University of Bern, Bern and Vientiane: Geographica Bernensia

[B44] Lao Women's Union, GRID Lao PDR. Gender Profile 2005 http://www-wds.worldbank.org/external/default/WDSContentServer/WDSP/IB/2009/01/02/000333037_20090102033411/Rendered/PDF/453750WP00BOX0334096B01PUBLIC1.pdf

[B45] Alvesson HM Lindelow M Khanthaphat B Laflamme L Shaping healthcare-seeking processes during fatal illness in resource-poor settings A study in Lao PDR. BMC Health Serv Res 2012 12 477 10.1186/1472-6963-12-477 PMC3543714 23259434

[B46] National Statistics Center Population Census 2005 2007 Vientiane, Lao PDR: National Statistics Center

[B47] Cross J MacGregor HN Knowledge, legitimacy and economic practice in informal markets for medicine: a critical review of research Soc Sci Med 2010 71 9 1593 1600 10.1016/j.socscimed.2010.07.040 20855143

[B48] Thorne S Reimer Kirkham S O'Flynn-Magee K The analytical challenge in interpretive description 2004 Methods: International Journal of Qualitative 3(1)

[B49] Thorne S Interpretive description 2008 Walnut Creek, CA: Left Coast Press Inc

[B50] Green J Thorogood N Qualitative Methods For Health Research 2009 London: Sage Publications Ltd

[B51] Patcharanarumol W Health care financing for the poor in Lao PDR 2008 London School of Hygiene and Tropical Medicine: Department of Public Health and Policy

[B52] Birungi H Injections and self-help: risk and trust in Ugandan health care Soc Sci Med 1998 4 10 1455 1462 9823041 10.1016/s0277-9536(98)00194-4

[B53] Patcharanarumol W Mills A Tangcharoensathien V Dealing with the cost of illness: the experience of four villages in Lao PDR J Int Dev 2009 21 2 212 230 10.1002/jid.1549

[B54] Ekman B Liem NT Duc HA Axelson H Health insurance reform in Vietnam: a review of recent developments and future challenges Health Policy Plan 2008 23 4 252 263 10.1093/heapol/czn009 18424793

[B55] Lee JY Kearns RA Friesen W Seeking affective health care: Korean immigrants' use of homeland medical services Health Place 2010 16 1 108 115 10.1016/j.healthplace.2009.09.003 19840903

[B56] Russell S Treatment-seeking behaviour in urban Sri Lanka: trusting the state, trusting private providers Soc Sci Med 2005 61 7 1396 1407 10.1016/j.socscimed.2004.11.077 16005775

[B57] Tibandebage P Mackintosh M The market shaping of charges, trust and abuse: health care transactions in Tanzania Soc Sci Med 2005 61 7 1385 1395 10.1016/j.socscimed.2004.11.072 16005774

[B58] Feierman S Kleinman A Stewart K Farmer D Das V Anthropology, knowledge-flows and global health Glob Public Health 2010 5 2 122 128 10.1080/17441690903401338 20013523

[B59] Grimen H Power, trust, and risk: some reflections on an absent issue Med Anthropol Q 2009 23 1 16 33 10.1111/j.1548-1387.2009.01035.x 19449710

[B60] Malqvist M Nga NT Eriksson L Wallin L Hoa DP Persson LA Ethnic inequity in neonatal survival: a case-referent study in northern Vietnam Acta Paediatr 2011 100 3 340 346 10.1111/j.1651-2227.2010.02065.x 20958789

[B61] Stenson B Syhakhang L Eriksson B Tomson G Real world pharmacy: assessing the quality of private pharmacy practice in the Lao People's Democratic Republic Soc Sci Med 2001 52 3 393 404 10.1016/S0277-9536(00)00142-8 11330774

[B62] World Bank Lao PDR. Civil Service Pay and Compensation Review: Attracting and Motivating Civil Servants 2010 Washington DC: World Bank

[B63] Hampshire KR Porter G Owusu SA Tanle A Abane A Out of the reach of children? Young people's health-seeking practices and agency in Africa's newly-emerging therapeutic landscapes Soc Sci Med 2011 73 5 702 710 10.1016/j.socscimed.2011.06.035 21824698

[B64] Bhutta ZA Ahmed T Black RE Cousens S Dewey K Giugliani E Haider BA Kirkwood B Morris SS Sachdev HPS What works? Interventions for maternal and child undernutrition and survival Lancet 2008 371 417 440 10.1016/S0140-6736(07)61693-6 18206226

[B65] World Bank Repositioning Nutrition as Central to Development 2006 Washington DC: World Bank

[B66] Makaula P Bloch P Banda HM Bongololo Mbera G Mangani C De Sousa A Nkhono E Jemu S Muula AS Primary health care in rural Malawi - a qualitative assessment exploring the relevance of the community-directed interventions approach BMC Health Serv Res 2012 12 1 328 10.1186/1472-6963-12-328 22995125 PMC3576236

[B67] Aubel J Toure I Diagne M Senegalese grandmothers promote improved maternal and child nutrition practices: the guardians of tradition are not averse to change Soc Sci Med 2004 59 5 945 959 10.1016/j.socscimed.2003.11.044 15186896

[B68] Sychareun V Hansana V Somphet V Xayavong S Phengsavanh A Popenoe R Reasons rural Laotians choose home deliveries over delivery at health facilities: a qualitative study BMC Pregnancy Childbirth 2012 12 1 86 10.1186/1471-2393-12-86 22925107 PMC3449206

[B69] Manithip C Quality and Utilisation of antenatal care services in rural Lao PDR 2012 Karolinska Institutet: Department of Women's and Children's Health

[B70] You D Wardlaw T Salama P Jones G Levels and trends in under-5 mortality, 1990–2008 Lancet 2010 375 9709 100 103 10.1016/S0140-6736(09)61601-9 19748116

[B71] Obrist B Iteba N Lengeler C Makemba A Mshana C Nathan R Alba S Dillip A Hetzel MW Mayumana I Access to health care in contexts of livelihood insecurity: a framework for analysis and action PLoS Med 2007 4 10 1584 1588 17958467 10.1371/journal.pmed.0040308 PMC2039761

[B72] Leonard KL Masatu MC Variations in the quality of care accessible to rural communities in Tanzania Health Aff (Millwood) 2007 26 3 w380 392 10.1377/hlthaff.26.3.w380 17389635

